# A Baseline Analysis of Regulatory Review Timelines for ANVISA: 2013–2016

**DOI:** 10.1007/s43441-020-00169-5

**Published:** 2020-06-09

**Authors:** Prisha Patel, Daniela Marreco Cerqueira, Gustavo Mendes Lima Santos, Renata de Lima Soares, Varley Dias Sousa, Lawrence Liberti, Neil McAuslane

**Affiliations:** 1 Centre for Innovation in Regulatory Science, 160 Blackfriars Road, London, SE1 8EZ UK; 2 Agência Nacional de Vigilância Sanitária, SIA Trecho 5, Área Especial 57, Brasília, DF CEP 71.205-050 Brazil

**Keywords:** Agência Nacional de Vigilância Sanitária (ANVISA), Regulatory review times, Optimizing Efficiencies in Regulatory Agencies (OpERA), Regulatory benchmarking

## Abstract

**Background:**

The Brazilian health regulatory agency (Agência Nacional de Vigilância Sanitária, ANVISA) has embarked on transformational initiatives to fulfill its mandate to provide timely access to safe, effective, and quality therapeutics. A new Brazilian law was enacted to provide the agency with greater flexibility. Optimizing Efficiencies in Regulatory Agencies (OpERA) is a regulatory-strengthening program that seeks to provide benchmarking data that can be used to define performance targets and focus performance improvement. The objective of this study was to use OpERA methodology to undertake a retrospective analysis of the timelines associated with important components of the ANVISA regulatory review process to establish a baseline against which the influence of the new law could be measured.

**Methods:**

The OpERA tool was used to collect specific milestone data that identify time periods, review stages, and data points for products approved by ANVISA 2013–2016.

**Results:**

For the 138 products approved in this cohort, the overall median approval time was 795 days. ANVISA and submitting companies will need to reduce their review and response times by approximately half in order to meet the total time goal of 365 days.

**Conclusions:**

The observations from this baseline study have identified opportunities for ANVISA and sponsor companies to collaborate to reduce regulatory assessment times while assuring the timely approval of safe and effective, quality medicines. These analyses will be repeated to determine how the provisions of the new Law will impact the activities of ANVISA and the extent of sponsors' contributions to this effort.

## Introduction

Measuring performance involves collecting and reporting data on practices, processes, and outcomes. Measuring pharmaceutical regulatory performance provides a necessary basis for a structured discussion with stakeholders to identify key indicators to monitor and improve processes. Integrating these indicators into regulatory practices by monitoring regulatory assessment times enables transparent tracking of process improvement initiatives [1]. This information can be used to identify and prioritize improvement goals and to track progress toward those goals and to monitor the maintenance of changes that have been already made. The first requirement of any performance measurement is to formulate a robust conceptual framework within which performance measures can be developed. Definitions of performance indicators should fit into the framework and satisfy several criteria, such as validity, reproducibility, acceptability, feasibility, and reliability [2].

The measurement of regulatory review performance should be documented and tracked to identify where time is spent in the regulatory process, thus ensuring the efficiency of this process as it evolves. This helps regulators and other stakeholders understand what drives regulatory review time and facilitates the integration of the practice of tracking and measuring regulatory performance, thereby promoting continuous improvement in review and approval times while ensuring safety, efficacy, and quality in medicine. Hence, the need for agencies to proactively and consistently measure their performance against stated target times is one of the World Health Organization (WHO) global benchmarking tool parameters [3].

## Brazil

With a current population of more than 209 million [4], Brazil’s gross domestic product (GDP) was 1.868 trillion USD in 2018 [5]. In 2016, healthcare expenditure represented 11.8% of Brazil’s GDP [6], and this is expected to grow, aided by increased government investment in the country’s universal and free public healthcare system, supporting programs to improve access to health services and medicines among all of its population.

Ranked among the world’s top ten largest pharmaceutical markets [7], all major pharmaceutical companies operate in Brazil [8] and the value of that market is forecast to grow to 29.9 billion USD in 2020 [7]. As recognized by former Minister of Health Ricardo Barros, “Brazil undoubtedly holds great development opportunities for the global pharmaceutical and healthcare industries, and we hope to gain the trust of an increasing number of international investors and jointly work on improving the healthcare products and services” [9].

## Agência Nacional de Vigilância Sanitária (ANVISA)

Established in 1999, ANVISA regulates medicinal products for human use, medical devices, food, cosmetics, and sanitizers. The total number of staff at ANVISA is approximately 1,600, including 200 reviewers of marketing authorization/product licenses, who are primarily pharmacists. The total annual budget of 840 million USD is 40% government funded and 60% fee based.

Underlining the agency’s important efforts to ensure the highest global standards against this background of rapid growth, former ANVISA Director/President Dr Barbosa da Silva stated that “ANVISA has conducted comprehensive review of its process, with the objective to strengthen the registration process, and to have a more transparent and predictable ecosystem for all stakeholders” [10]. In fact, ANVISA has embarked on several transformational initiatives to ensure that it will continue to be in a strong position to fulfill its mandate of providing timely access to safe, effective, and quality therapeutics. The agency is a regulatory member of the International Council of Harmonization of Technical Requirements for Pharmaceuticals for Human Use (ICH) since 2016 and was accepted as member of the Management Committee in 2019. ANVISA is also recognized as a Level IV reference agency by the Pan American Health Organization (PAHO) and has entered into a variety of international collaborative agreements such as the Statement of Cooperation (SOC) with the US Food and Drug Administration (US FDA) that is intended to strengthen existing structures and develop new opportunities for cooperative engagement in regulatory and scientific matters and public health protection.

However, because of its broad mandate to address the ongoing assessment of a wide variety of medicinal products, manpower limitations, and the need to work within the legal framework for conducting regulatory reviews, the agency had been faced with regulatory review timelines that were among the longest for Latin American countries [11]. In addition, other factors such as the obligation to perform full reviews, protracted company response times, and the requirement for a Certificate of Pharmaceutical Product for product approval contribute to lengthy review times. Prolonged regulatory timelines have been a limitation to patient access to medicines [12].

In response to these issues, in December 2016, the new Law Number 13,411 was enacted to modify existing legislation to provide the agency with greater flexibility in its approaches to medicine regulation. Among the important innovations of this new law, which went into effect in March 2017, is a risk-based approach addressing the technical complexity of products. In addition, this law specifies the clinical, economic, and social benefits of the medication that determine its status as regulatory review category I—a priority medicines, for which reviews are to be conducted in 120 days of receipt of the marketing authorization application (MAA) or category II—an ordinary medicine, for which reviews are to be conducted within 365 days of MAA receipt. It should be noted that the timelines may be extended by up to one-third of the original deadline. Also, ANVISA requests for clarification or rectification suspend these deadlines until company responses are received, which must be within 120 days of the agency request.

Recognizing that the new law could have a positive impact on workload, efficiency, and ultimately, process times, ANVISA collaborated with the Centre for Innovation in Regulatory Science (CIRS, www.cirsci.org) to undertake a retrospective analysis of the timelines associated with important components of the ANVISA regulatory review process to establish a baseline against which the influence of the new law could be measured. This study represents the first comprehensive analysis of ANVISA regulatory activity timelines (addressing both agency and company time) across multiple years, product types, and therapeutic areas.

## Methodology

CIRS has been collaborating with regulators from around the world to develop the bespoke program entitled “Optimizing Efficiencies in Regulatory Agencies” (OpERA). OpERA is a multi-year project initiated by CIRS in 2013 based on requests from regulatory agencies. Objectives of the program are to (1) provide benchmarking data that can be used to define performance targets and focus ongoing performance improvement initiatives, (2) accurately compare the processes used in the review of new drug marketing authorizations, (3) encourage the sharing of information on common practices in order to learn from others’ experiences, and (4) encourage systematic measuring of the processes that occur during the review of new drug marketing authorizations [13].

The OpERA methodology comprises two components: a process assessment analysis designed to clearly assess the component activities associated with the medicine review and assessment processes within an agency or regional regulatory initiative (RRI) and the collection of key milestone metrics aligned with the elements of the process assessment. The specific milestones include time periods, review stages, and data points that have been selected by agencies and RRIs participating in the OpERA program so as to permit a detailed analysis of an agency’s efficiency (Table 1).

**Table 1.** Key Review Milestones Monitored.

Key Milestone Dates
1a. Receipt of the dossier
1b. Acceptance to file
2a. Start of Primary Scientific Assessment
2b. Completion of Primary Scientific Assessment Primary Scientific Assessment
3a. Primary assessment deficiency letter sent to sponsor (if applicable)
3b. Response from Sponsor (If applicable)
4. Secondary assessment following deficiency letter response (if applicable)
5. Advisory Committee Review (if applicable)
6. Completion of Scientific Assessment
7. Marketing Authorization Granted/Rejected

For REC: Final Acceptance by member state

Participating agencies and RRIs have identified commonly collected milestones that demonstrate both the agency and company time associated with the medicine review process. Results obtained from OpERA analysis help agencies identify where time is spent in their processes, define and meet their regulatory performance goals, monitor change activities, embed a culture of ongoing self-assessment, optimize their process efficiencies, and increase internal/external transparency.

ANVISA provided to CIRS product characteristics and regulatory milestone dates consistent with those collected through the OpERA program. This analysis focused on products approved by ANVISA between January 1, 2013 and December 31, 2016.

Assessments were conducted for new active substances (NASs), major line extensions (MLEs), biologics, and generics. An Anatomical, Therapeutic, Chemical (ATC) category was assigned to each product by ANVISA. All products were anonymized using a random coding assigned by ANVISA prior to submitting the data to CIRS.

Data were provided by ANVISA in Microsoft Excel for the following milestones: Receipt of the dossier (Dossier validation); Start of primary scientific assessment; Completion of primary scientific assessment (Primary scientific assessment); Primary assessment deficiency letter sent to sponsor; Response from sponsor (If applicable) (Clock stop/sponsor time); Additional cycles of assessment following deficiency letter response (if applicable) (Secondary scientific assessments); Advisory Committee review (if applicable) (Advisory Committee); Marketing authorization granted. A product could have undergone multiple review cycles. Data were checked for consistency and completeness by CIRS and clarifications were provided by ANVISA.

Timelines in calendar days were calculated for the following sequences: receipt of dossier to start of primary assessment (which includes queue time and dossier validation); start of scientific assessment to end of first scientific assessment (primary scientific assessment); outcome letter 1 response received from sponsor (sponsor time); response of outcome letter to end of scientific assessment (subsequent scientific assessments); advisory committee time (if relevant); response from outcome letter to decision of MAA (overall approval time).

In a move to reduce review backlogs, in 2013 sponsors of generic products were offered a one-time opportunity to advance selected products to an earlier position in the review queue. This “switch” opportunity has been reflected in these analyses; for these 86 products, the switch date of April 15, 2013 has been used as the date for the receipt of the submission. All analyses of generic products were conducted after adjustment for switch dates.

## Results

### Data Set Characteristics

During the time period of 2013 to 2016, 235 products were submitted for regulatory review (Table 2): these represented 30 NASs (13%), 16 MLEs (7%), 25 biologics (11%), and 164 generics (70%). By December 31, 2016, regulatory approvals had been given to 138 products comprising 20 NASs (14%), 9 (7%) MLEs, 19 (14%) biologics, and 90 (65%) generics. Not all products submitted from 2013 to 2016 were approved by ANVISA.

**Table 2.** Number of Products Submitted to and Approved by ANVISA by Product Type, 2013–2016 (*N* = 235).

Compound Types	Products Approved 2016, *n* (% of Total)	Products Submitted 2013, *n* (% of Total)	Products Submitted 2014, *n* (% of Total)	Products Submitted 2015, *n* (% of Total)	Products Submitted 2016, *n* (% of Total)	Products Submitted 2013–2016, *n* (% of Total)
Generics	90 (65%)	106 (88%)	24 (50%)	26 (54%)	8 (44%)	164 (70%)
New active substances	20 (14%)	5 (4%)	11 (23%)	12 (25%)	2 (11%)	30 (13%)
Biologics	19 (14%)	1 (0.8%)	10 (21%)	6 (13%)	8 (44%)	25 (11%)
Major line extensions	9 (7%)	9 (7%)	3 (6%)	4 (8%)	0	16 (7%)
Totals	138	121	48	48	18	235

The numbers of products submitted by year are detailed in Table 2. Because of the generic switch opportunity, 2013 saw the most submissions. For the 235 submitted products, the most common therapeutic areas were nervous system (46 (20%), cardiovascular (32 (14%) and anticancer/immunomodulators (28 (12%).

The 46 products submitted by multinational pharmaceutical companies accounted for the majority of NASs and biologic approvals. Local (Brazilian) companies submitted 189 products, representing the vast majority of MLEs and generic submissions. Consequently, approvals from 2013 to 2016 comprised 103 products from local companies and 35 from multinationals.

### Regulatory Timing Metrics

**Figure 1.**
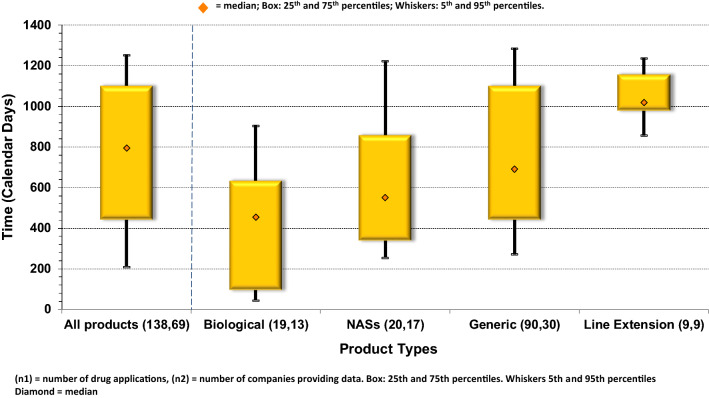
Regulatory approval times (agency and company time) for products approvals through 2016.

For the 138 products approved in this cohort, the overall median approval time was 795 days; this comprised median review times by product type of 691 days (generics), 552 days (NASs), 454 days (biologics), and 1,018 days (MLEs). The widest variability (25th to 75th percentiles) in approval times was observed for generics (653 days) while the narrowest variance was for MLEs (172 days) (Fig. 1).

**Figure 2.**
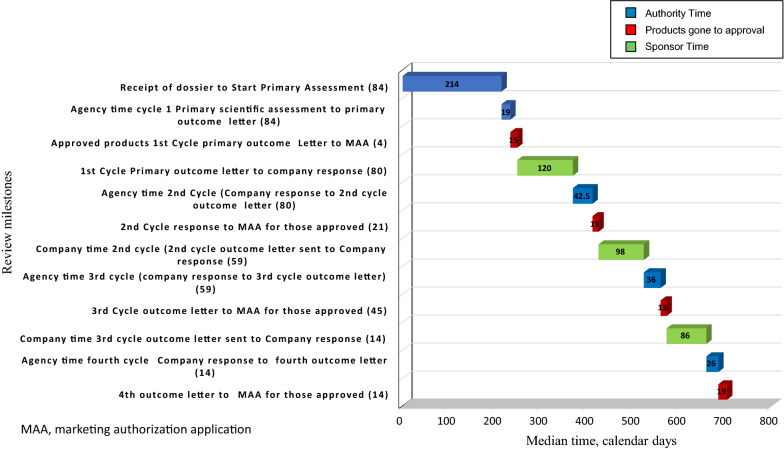
Assessment of regulatory activity timelines: products approved by December 31 2016.

An analysis of the review process was conducted to identify the time taken for each cycle review (Fig. 2). The median time between each milestone for standard review compounds submitted to ANVISA between 2013 and 2016 was calculated. Because of some missing milestone data, criteria were applied for the application to either be excluded from this analysis or included through the extrapolation of other available data.

Specifically, the following types of applications were excluded from the analysis:

- where there was no “start of primary scientific assessment”;
- where there was no “company response date” or “start of scientific assessment” date;
- where there was no “completion of scientific assessment” or “outcome letter sent date”.

The following applications were included in the analysis with the use of substitute data:

- Where “no outcome letter sent” date was provided, the “completion of scientific assessment” date was used.
- Where “completion of scientific assessment” date was provided but no “outcome letter sent” date was provided, the “completion of scientific assessment” date was used for the “date the outcome letter sent”.
- Where there was a no “company response” date but a “start of scientific assessment” date was given, the “start of scientific assessment” date was used.
- Where there was no “completion of scientific assessment” date, and there are no further cycles, the “completion of all scientific assessment” date was used as the end date of that cycle, if this was within 30–40 days of the start of that cycle.

In addition, other types of applications excluded from analysis included the following:

- applications rejected by ANVISA;
- applications that had more than four cycles of review, which were considered as special cases and not the usual review process for ANVISA;
- applications that were in the appeal process, but stayed in the review system, until the appeal decision was made;
- applications where the date of “start of scientific assessment” or “company response” date for a cycle was present but no other information except date of “completion of all scientific assessment”; if this was longer than 30-40 days, the application was rejected from the analysis as it was not clear if this was company or agency time.

Using these criteria, a total of 84 applications were analyzed. Most of the approved products underwent a 3-cycle review. Four applications (5%) were approved in the first cycle, 21 (25%) in the second cycle, 45 (54%) in the third cycle, and 14 (17%) in the fourth cycle. The overall review time for 84 applications was 684 median days. For the majority of applications, which went through 3-cycle reviews, the median approval time was 557 median days. The majority of agency time was between receipt of dossier to start of primary assessment. The company time ranged from 86 to 120 median days (Fig. 2). Table 3 shows the variance of each milestone for the 5th and 95th percentile.

**Table 3.** Milestone Timing and Variance.

Review Milestone	Median Time, Calendar Days	5th Percentile, Calendar Days	95th Percentile, Calendar Days
Receipt of dossier to Start primary assessment (84)	214	28	989
Agency time 1st cycle Primary scientific assessment to Primary outcome letter (84)	19	3	168
Approved products 1st Cycle Primary outcome letter to MAA (4)	15	8	42
1st Cycle primary outcome letter to Company response (80)	120	21	236
Agency time 2nd Cycle Company response to 2nd cycle outcome letter (80)	43	13	141
2nd Cycle Response to MAA for those approved (21)	13	6	42
Company time 2nd cycle 2nd cycle outcome letter sent to Company response (59)	98	7	125
Agency time 3rd cycle Company response to 3rd cycle outcome letter (59)	36	5	114
3rd Cycle outcome letter to MAA for those approved (45)	13	6	32
Company time 3rd cycle outcome letter sent to Company response (14)	86	8	127
Agency time fourth cycle Company response to fourth outcome letter (14)	26	7	67
4th outcome letter to MAA for those approved (14)	19	6	49

*MAA* marketing authorisation application.

When assessed by therapeutic area, for NASs submitted between 2013 and 2016, hormone therapies had the shortest median review time (733 days) compared with dermatologic products (median, 1512 days). Anticancer NASs had a median review time of 1312 days. Median review times for MLEs ranged from 983 days (nervous system therapies) to 2320 days (blood products) and for biologics from 70 days (musculoskeletal products) to 787 days (immunomodulators). Median review times for generics ranged from 266 days (dermatologic products) to 1688 (respiratory products).

## Discussion

These observations represent an important analysis of ANVISA regulatory activity timelines (addressing both agency and company time) across multiple years, product types, and therapeutic areas. Under the terms of Law Number 13,411, individual reviewers may be held liable for non-compliance with the stated timelines. While this degree of personal liability is not observed frequently in mature regulatory agencies, it may present a challenge to reviewers faced with the assessment of complex NASs or biologic products.

ANVISA have developed and implemented an assessment template for the review of the safety and efficacy of medicines. The template includes critical questions for the assessor to ask during the review, including reference documents to support the review. The introduction of the template will provide transparency, consistency, and compliance with the timelines.

The target total time for ANVISA registration (agency and company time) is up to 365 days. In this study, the median agency time was 389 median days and company time was 304 median days. This indicates that the agency is close to meeting the total time goal of 365 days established by Law 13,411.

Our observations indicate that a significant time savings can be obtained by reducing the time from receipt of the dossier to the time of the start of the first scientific assessment (214-day queue time), which occurred as the result of manpower limitations to start the scientific assessment. Should this manpower be increased, this time period could be used to validate the content of the dossier. This process is observed in some other agencies such as the European Medicines Agency. During this period a rapid validation (e.g., in under 2 weeks), requesting missing items could be conducted. With this process, the observed 15-day median for the first scientific assessment would likely be increased but this would be offset by a significantly shorter time to the start of the first assessment.

A quality and timely regulatory review is facilitated by a quality regulatory submission. Sponsors need to provide dossiers that reflect the needs and expectations of the sponsor. In order to further improve the time for patient access to medicines, sponsors should strive to respond to the agency in a timely manner. Company time represented one quarter (304 days) of the total approval time (684 days) across all approved products for this cohort. Company time can be influenced by a variety of factors including prioritization of products in a global regulatory environment, local capabilities to respond efficiently to ANVISA requests, and the nature of the clarifications required by the agency based on the initial quality of the submission. To streamline responses, the requests for major clarification or rectification by the agency are now being consolidated into a single request for each major dossier section, except when they are needed to clarify or rectify information related to a requirement previously answered by the applicant company.

In 2017 and 2018, ANVISA published three new resolutions with the purpose of accelerating the approval of medicines; Resolution 204/2017, Resolution 205/2017, and Service Orientation 45/2018. Resolution 204/2017 establishes “Priority Review” criteria for products that meet at least one of the eligibility criteria, for example, medicines for neglected diseases, and vaccines to be incorporated in the national immunization program. This guidance also addresses priority review processes for post-approval applications when there is a public health risk of drug shortages. In 2018, 173 applications were approved out of 827. The timeline for the final decision is 120 calendar days (365 calendar days for ordinary category) [14]. Resolution 205/2017 establishes a special procedure for the consent of clinical trials, certification of GMP, and registration of new medicines for treatment, diagnosis, or prevention of rare diseases. In 2018, the median timeline for the final decisions was 155 days for medicines evaluated under this resolution.

Service Orientation 45, which establishes optimized review for registration and post-registration changes for biological products, is being considered a “Reliance Pilot Project.” Products already approved by the US FDA and European Medicines Agency with same indications, dosage, adverse reactions, and precautions are eligible. Applicants must submit reports containing the criteria used by both agencies to review and approve these applications.

ANVISA also recognized the backlog of generic applications and has worked with international institutions such as CIRS to implement standardized risk assessment models to speed up the registration process for generics. Leveraging this regulatory update, it was possible for ANVISA to reduce the number of these registration files [10].

The new Brazilian law provides ANVISA with a degree of flexibility in addressing its approaches to regulatory reviews. One approach that is being used successfully by emerging agencies worldwide addresses submissions from a risk-based approach [15]. In these models, a product’s risk is assessed by various criteria established by the agency such as the number of agencies that have conducted a prior assessment of the product, whether they are considered reference agencies, or how long the product has been on the market. As ANVISA implements a reliance mechanism in which prior decisions can be used as the basis for informing the assessment, but wherein the agency retains the role of conducting a targeted benefit-risk assessment relevant to the Brazilian population, the efficient use of agency resources can be addressed while allowing the reviewers to maintain their ability to apply their expertise to the country-specific issues of the product [16].

## Data Limitations

Where there were missing data, datapoints were either substituted or excluded (as outlined above). Where no “company response” date was given, the date of “start of scientific assessment” was used; the company may have responded in a timely manner, but the date was not logged by the agency. As a result, the company time may have been overestimated. Agency time may have been underestimated with regards to a product’s last review cycle. If there is was no “completion of scientific assessment” date for that cycle, and there and there were no further cycles, the “completion of all scientific assessment” date was used as the end date of that cycle. If this time period was greater than 40 days, the datapoints were excluded, because of uncertainty around agency and company factors that may have had an impact. Even though caveats were applied, Fig. 2 still reflects the elements of the review process to achieve marketing authorization within ANVISA.

## Conclusions

The observations from this baseline study have identified possible opportunities for ANVISA and sponsor companies to collaborate to reduce regulatory assessment times while assuring the timely approval of safe and effective, quality medicines. These analyses will be repeated on a periodic basis to determine how the provisions of Law 13,411 will impact the activities of ANVISA and the extent to which the sponsors have maximized their contributions to this effort.
